# Predictors and prognosis of paroxysmal atrial fibrillation in general practice in the UK

**DOI:** 10.1186/1471-2261-5-20

**Published:** 2005-07-11

**Authors:** Ana Ruigómez, Saga Johansson, Mari-Ann Wallander, Luis Alberto García Rodríguez

**Affiliations:** 1 Centro Español de Investigación Farmacoepidemiológica (CEIFE), Madrid, Spain; 2 AstraZeneca R&D Mölndal, Sweden; 3 Section of Preventive Cardiology, Göteborg University, Sweden; 4 Department of Public Health and Caring Science, Uppsala University, Sweden

## Abstract

**Background:**

Natural history of paroxysmal atrial fibrillation (AF) is not very well documented. Clinical experience suggests that paroxysmal AF could progress to chronic AF with estimates ranging between 15 and 30% over a period of 1–3 years. We performed an epidemiologic study to elucidate the natural history of paroxysmal AF, this study estimated its incidence in a general practice setting, identified associated factors and analyzed the progression into chronic AF as well as the mortality rate.

**Methods:**

Using the UK General Practice Research Database (GPRD), we identified patients aged 40–89 years with a first-recorded episode of paroxysmal AF during 1996. Risk factors were assessed using 525 incident paroxysmal AF cases confirmed by the general practitioner (GP) and a random sample of controls. We follow-up paroxysmal AF patients and estimated their mortality rate and progression to chronic AF.

**Results:**

The incidence of paroxysmal AF was 1.0 per 1,000 person-years. Major risk factors for paroxysmal AF were age and prior valvular heart disease, ischaemic heart disease, heart failure and hyperthyroidism. During a mean follow-up of 2.7 years, 70 of 418 paroxysmal AF patients with complete information progressed to chronic AF. Risk factors associated with progression were valvular heart disease (OR 2.7, 95% CI 1.2–6.0) and moderate to high alcohol consumption (OR 3.0, 95% CI 1.1–8.0). Paroxysmal AF patients did not carry an increased risk of mortality, compared to an age and sex matched sample of the general population. There was a suggestion of a small increased risk among patients progressing to chronic AF (RR 1.5, 96% CI 0.8–2.9).

**Conclusion:**

Paroxysmal AF is a common arrhythmia in the general practice setting, increasing with age and commonly associated with other heart diseases. It sometimes is the initial presentation and then progress to chronic AF. A history of valvular heart disease and alcohol consumption are associated with this progression.

## Background

The incidence and natural history of paroxysmal atrial fibrillation (AF) have not been well studied[1]. Paroxysmal AF is usually defined temporally as intermittent periods of AF intertwined with episodes of normal sinus rhythm, normally lasting less than a week [2,3]. It could be a unique self-terminating episode of AF or recurrent ones [4]. When the arrhythmia is sustained in time, AF is designated persistent or chronic AF. However, the differentiation of paroxysmal AF from more chronic forms of AF is often based on the history given by symptomatic patients. This could be misleading, as asymptomatic paroxysmal AF is quite common [5,6]. Paroxysmal AF may be the initial presentation of the arrhythmia or can continue with recurrent episodes that may eventually become chronic as the end result AF[4,7,8]. Some reports have shown that about 25% of paroxysmal AF patients will develop a more persistent form of AF [3]. The rate of transition from paroxysmal to chronic varies considerably with the underlying aetiology, being more frequent among patients with rheumatic heart disease and ischaemic heart disease. Progression from paroxysmal to chronic form does not depend on age or gender but on longer duration of the initial paroxysmal AF episode [8,9].

Most of the studies published on atrial fibrillation patients are based on hospital patients, or cohorts of patients undergoing specific clinical diagnostic tests and procedures. We performed an epidemiologic study in the general practice setting using information recorded by the general practitioners during their daily consultations.

The objectives of this study were to estimate the incidence rate of paroxysmal AF as compared with chronic AF in a general practice setting [10], to describe comorbidity and health care utilization and to identify factors associated with paroxysmal occurrence. We also estimated the rate of progression to chronic AF, as well as the mortality rate among patients with first-detected paroxysmal AF.

## Methods

We performed a cohort study using data from the General Practice Research Database (GPRD). The general practitioners (GPs) who anonymously provide the GPRD with their data systematically record information on demographics, medical diagnoses, referrals to consultants and hospitals, and written prescriptions for their patients. The database contains computerized information for more than two million residents in the UK registered with GPs and is administered by the Medicines and Healthcare products Regulatory Agency (MHRA), which organizes this information to be used for research projects. Numerous epidemiological studies support its validity and data completeness, including a recent study on AF [11,12].

### Study population

We identified a total of 703,777 patients in the GPRD meeting the following conditions during 1996. Patients were 40–89 years old, enrolled with a GP for more than 2 years and with at least one health contact before January 1996. These eligibility criteria were applied to ensure that all study members had incurrred in some recent contact with the GP prior to entering the study period and were then at risk of being cared "actively" by their GP. Patients with a code for heart rhythm disorders (ICD 8th edition: 4160–4169) or cancer (ICD 8th: 1400–2099) before January 1996 were omitted.

### Case definition and validation

We identified 2,098 patients attending to the general practitioner with a first-ever recorded diagnosis of AF/flutter (ICD 8th: 4163). After reviewing the computerized records for all of them, 1,972 patients were considered potential cases of AF. For all of them a questionnaire was sent to the GPs requesting additional information. Additionally, 623 patients with unspecific codes of supra-ventricular and sinus arrhythmias other than AF were identified. Since these codes could mask a diagnosis of AF, we sent a questionnaire to an approximately 10% random sample (n = 68). The GP was asked to confirm the diagnosis of AF, and to classify it as chronic or paroxysmal based on the following criteria: Chronic AF was defined as persistence of the arrhythmia, with the episode of AF not converting to sinus rhythm within 1 week (this definition include persistent and permanent AF cases on terminology proposed by the ACC/AHA/ESC guidelines) [4]. We considered paroxysmal AF when the arrhythmia did revert spontaneously or following treatment to sinus rhythm within a week. We also requested the GPs to confirm whether this diagnosis was the first ever and to provide information on diagnostic tests, procedures and aetiology of the AF. Patient confidentiality was always preserved.

We received 1,888 valid questionnaires (95% response rate). Five hundred twenty-five patients were confirmed as paroxysmal AF cases and 1,109 were patients with chronic AF. The remaining patients were not confirmed as having AF (n = 125) or had an episode of AF before entering the study period (n = 129). The confirmation rate was 98% among patients originally identified with AF codes and 30% among those with unspecific codes of arrhythmia. Patients with chronic forms of AF were analysed separately, and the main results have been published recently [10,12].

### Incidence analysis

We estimated the incidence rate of paroxysmal AF stratified by age groups and sex. We used confirmed incident cases of paroxysmal AF as the numerator and the sum of person-years in the study population as the denominator within age and sex strata.

### Nested case-control analysis to ascertain risk factor for paroxysmal AF

We used all confirmed cases of paroxysmal AF (as in the cohort analysis), and their index date was the date of the paroxysmal AF diagnosis. We then assigned a random date between 1 January and 31 December 1996 to all members of the study population. If this random date was included in her/his contribution of person-time to the study, that person became an eligible control and we used that date as index date. Finally, 5,000 controls were randomly sampled from the pool of eligible controls. This selection process warrants that the likelihood of being selected as a control is proportional to the person-time at risk.

We used the information recorded by the GP in the computerized database for both cases and controls to ascertain demographic data, as well as the prevalence of medical history before index date for the following conditions: ischaemic heart disease (IHD), valvular disease, heart failure (HF), hypertension, cerebrovascular disease (CVD), diabetes and hyperthyroidism. We also ascertained the role of smoking status, body mass index (BMI) and alcohol consumption. We computed estimates of the odds ratio (OR) and 95% confidence interval (CI) of AF associated with risk factors, using unconditional logistic regression adjusted by age and sex.

### Mortality follow-up analysis

Using the study population in which AF patients were identified and applying the same eligibility criteria as for the AF cohort, we randomly sampled a cohort of 5,000 individuals free of AF, matched to the cohort of paroxysmal AF patients by age and sex. The paroxysmal AF cohort was followed up from date of AF diagnosis and the general population cohort from a random date during 1996 until the earlier of either death or end of follow-up (April 2001). Survival probability was computed in both cohorts, and we estimated the relative risk (RR) of mortality associated with AF, using Cox proportional hazard regression. We retrieved death information from computerized files and from the questionnaire filled by the GP, and ascertained the cause of death using the two sources of information.

### Follow-up analysis for progression to chronic AF

In order to assess which paroxysmal AF patients progressed to chronic AF, we sent a second questionnaire to the GPs requesting depersonalised copies of medical records for all paroxysmal AF patients. At the time of this second request, we received valid information on 418 cases; the remaining patients (n = 107) were no longer reachable. The GPs confirmed that 70 patients (17%) developed chonic AF before April 2001, 192 continued with recurrent episodes of paroxysmal AF and the remaining (n = 156) did not present any more episodes after the first one.

We used a life table analysis to show the proportion of patients with initial paroxysmal AF, that progressed to chronic AF during the follow-up period.

A nested case-control analysis was performed among the 418 paroxysmal AF patients with valid information to assess risk factors for progression to chronic forms. In this analysis, we used as cases the 70 patients who progressed to chronic AF, and we used their date of diagnosis of chronic AF as index date. All remaining AF patients (n = 348) were used as controls. Estimates of progression risk and 95% CI were computed using unconditional logistic regression. We collected recorded information on the following risk factors: smoking status, BMI and alcohol consumption, as well as prior history of HF, valvular disease, IHD, CVD, hypertension and diabetes. We assessed the association between drug treatment and the development of persistent AF. We also estimated the mortality rate in both groups: those who had paroxysmal AF and those who developed chronic AF.

## Results

**Figure 1:**
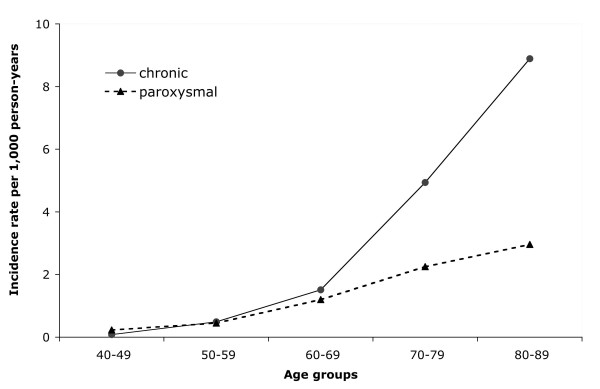
Incidence of paroxysmal atrial fibrillation in comparison to chronic atrial fibrillation in UK General Practice [10].

The incidence rate of paroxysmal AF was 1.0 per 1000 person-years (95% CI 0.9–1.1). In a previous reported study on the same source population we found that the incidence of chronic AFwas higher (1.7, 95% CI 1.6–1.8) [10]. The incidence increased with age in both AF groups, but this was more pronounced among persons aged 70 years and older in the cohort of chronic AF, compared with the paroxysmal AF cohort (Fig. 1). The incidence of paroxysmal AF was similar in males and females.

Characteristics of the 525 patients with incident paroxysmal AF are shown in Table 1. The mean age at presentation was significantly higher in the female population (73 years; SD = 10) than in the male population (67 years; SD = 11). The most frequent aetiology among initially detected paroxysmal AF as assigned by the GP was IHD in both sexes (43%), while no specific cause was given in 32% of the cases. Less than a third of patients with an initial paroxysmal AF episode had a cardioversion attempt close to the time of diagnosis. Digoxin and beta-blockers were the most frequently prescribed drugs during the 3 months after the paroxysmal AF episode. Close to half of the paroxysmal AF patients (48%) did not receive warfarin or aspirin during the first 3 months after diagnosis.

**Table 1:** Distribution of age, aetiology, diagnostic tests and pattern of treatment among paroxysmal atrial fibrillation patients by sex

	Female		Male	
	n = 268	(%)	n = 257	(%)
	
**Age**				
40–59	30	(11.2)	63	(24.5)
60–69	57	(21.3)	74	(28.8)
70–79	107	(39.9)	81	(31.5)
80+	74	(27.6)	39	(15.2)
AF aetiology assigned by the GP*				
IHD	117	(43.7)	110	(42.8)
Valvular	24	(9.0)	13	(5.1)
Other cardiac diseases	19	(7.1)	20	(7.8)
Non-cardiac diseases	28	(10.4)	16	(10.1)
Unknown	80	(29.9)	88	(34.2)
**Tests done to confirm diagnosis**				
ECG alone	158	(59.0)	170	(66.1)
Other test (with/without ECG)	46	(17.1)	33	(12.8)
Unknown	64	(23.9)	54	(21.0)
Cardioversion attempts				
No or unknown	178	(66.4)	170	(66.2)
Pharmacological only	79	(29.5)	69	(26.8)
Electrical only	8	(3.0)	13	(5.1)
Both	3	(1.1)	5	(1.9)
**Use of AF treatment drugs †**				
Amiodarone	27	(10.1)	40	(15.6)
Verapamil	8	(3.0)	7	(2.7)
Diltiazem	13	(4.9)	13	(5.1)
Beta-blockers	82	(30.6)	65	(25.3)
Digoxin	100	(37.3)	80	(31.1)
**Use of antithrombotics/anticoagulants†**				
No use	136	(50.7)	115	(44.7)
Warfarin only	28	(10.4)	36	(14.0)
Aspirin only	92	(34.3)	91	(35.4)
Both	12	(4.5)	15	(5.8)

*17 cases (3.2%) had the episode after a cardiovascular surgery.

†In the first 3 months after initial diagnosis.

Baseline characteristics of paroxysmal AF patients and controls are shown in Table 2. Age was the most important risk factor for paroxysmal AF. Individuals 70 years old or more presented a relative risk greater than eight-fold of developing paroxysmal AF after adjustment for cardiovascular comorbidity and other risk factors. Male patients and those with a moderate to high alcohol consumption carried an increased risk of paroxysmal AF.

**Table 2:** Risk of paroxysmal atrial fibrillation associated with age, sex, and other factors

	Paroxysmal AF cases		Controls			
	n = 525	(%)	n = 5000	(%)	OR*	(95% CI)
	
**Age**						
40–49	31	(5.9)	1565	(31.3)	1	
50–59	62	(11.8)	1343	(26.9)	2.1	(1.3–3.2)
60–69	131	(25.0)	997	(19.9)	5.0	(3.3–7.5)
70–79	188	(35.8)	769	(15.4)	8.3	(5.5–12.5)
80–89	113	(21.5)	326	(6.5)	10.9	(7–17.1)
**Sex**						
Female	268	(51.0)	2647	(52.9)	1	
Male	257	(49.0)	2353	(47.1)	1.3	(1.0–1.6)
**Smoking†**						
Non-smoker	313	(59.6)	2736	(54.7)	1	
Smoker	94	(17.9)	1226	(24.5)	0.8	(0.6–1.0)
Ex-smoker	42	(8.0)	280	(5.6)	1.0	(0.7–1.5)
**Body mass index (BMI) †**						
<20	25	(4.8)	201	(4.0)	1.4	(0.9–2.4)
20–24	135	(25.7)	1466	(29.3)	1	
25–29	162	(30.9)	1420	(28.4)	1.1	(0.8–1.4)
30+	62	(11.8)	601	(12.0)	1.1	(0.8–1.5)
**Alcohol consumption (units per week) †#**						
None	185	(35.2)	1597	(31.9)	1	
1–7 units	114	(21.7)	1112	(22.2)	1.2	(0.9–1.6)
8–21 units	69	(13.1)	718	(14.4)	1.4	(1.0–1.9)
>21 units	31	(5.9)	292	(5.8)	1.7	(1.1–2.6)
**Comorbidity**						
IHD	156	(29.7)	415	(8.3)	2.1	(1.6–2.6)
Valvular disease	30	(5.7)	41	(0.8)	4.2	(2.4–7.3)
Heart failure	81	(15.4)	117	(2.3)	2.5	(1.8–3.5)
Hypertension	197	(37.5)	859	(17.2)	1.4	(1.2–1.8)
Cerebrovascular disease	68	(13.0)	195	(3.9)	1.5	(1.1–2.1)
Diabetes	36	(6.9)	194	(3.9)	0.9	(0.6–1.4)
Hyperthyroidism	19	(3.6)	44	(0.9)	3.6	(2.0–6.5)

*OR: Odds ratio adjusted by age, sex and all the variables in the table, using unconditional logistic regression.

†Missing data in smoking (15.1%), BMI (26.3%) and alcohol use (25.5%).

# 1 unit = 10 mL of pure ethanol.

Close to 70% of paroxysmal AF patients presented some cardiovascular morbidity compared with 29% of controls. Hypertension and IHD were the most prevalent diagnoses in both groups (Table 2). Patients with underlying valvulopathies had a four-fold increased risk of having paroxysmal AF. Other conditions independently associated with the development of paroxysmal AF were heart failure (OR 2.5; 95% CI 1.8–3.5) and hyperthyroidism (OR 3.6; 95% CI 2.0–6.5). We did not find any major association with diabetes.

During a mean follow-up period of 2.7 years, 67 paroxysmal AF patients died. The mortality rate among paroxysmal AF patients was 4.2 per 100 person-years. The relative risk of mortality among paroxysmal AF patients was similar to that in an age and sex matched sample of the general population free of AF after adjustement for other co-comorbidity (table 3). The most frequent cause of death was heart disease including IHD (46.3%), followed by cancer (13.4%). In nine cases, no specific cause of death was assigned.

**Table 3:** Mortality rate and relative risk of death associated with paroxysmal atrial fibrillation

	Age and sex matched Cohort free of AF n = 5000	Paroxysmal AF cohort n = 525
Person-years	14298	1606
Deaths	483	67
Mortality rate/100 person-years (95% CI)	3.38 (3.09–3.69)	4.17 (3.30–5.26)
Relative risk (95% CI)	1	1.2 (1.0–1.6)
Adjusted relative risk*(95% CI)	1	1.0 (0.75–1.3)

*Relative risk estimated by Cox regression model, including age, sex, smoking, heart failure, ischaemic heart disease, hypertension, cerebrovascular disease and diabetes.

**Figure 2:**
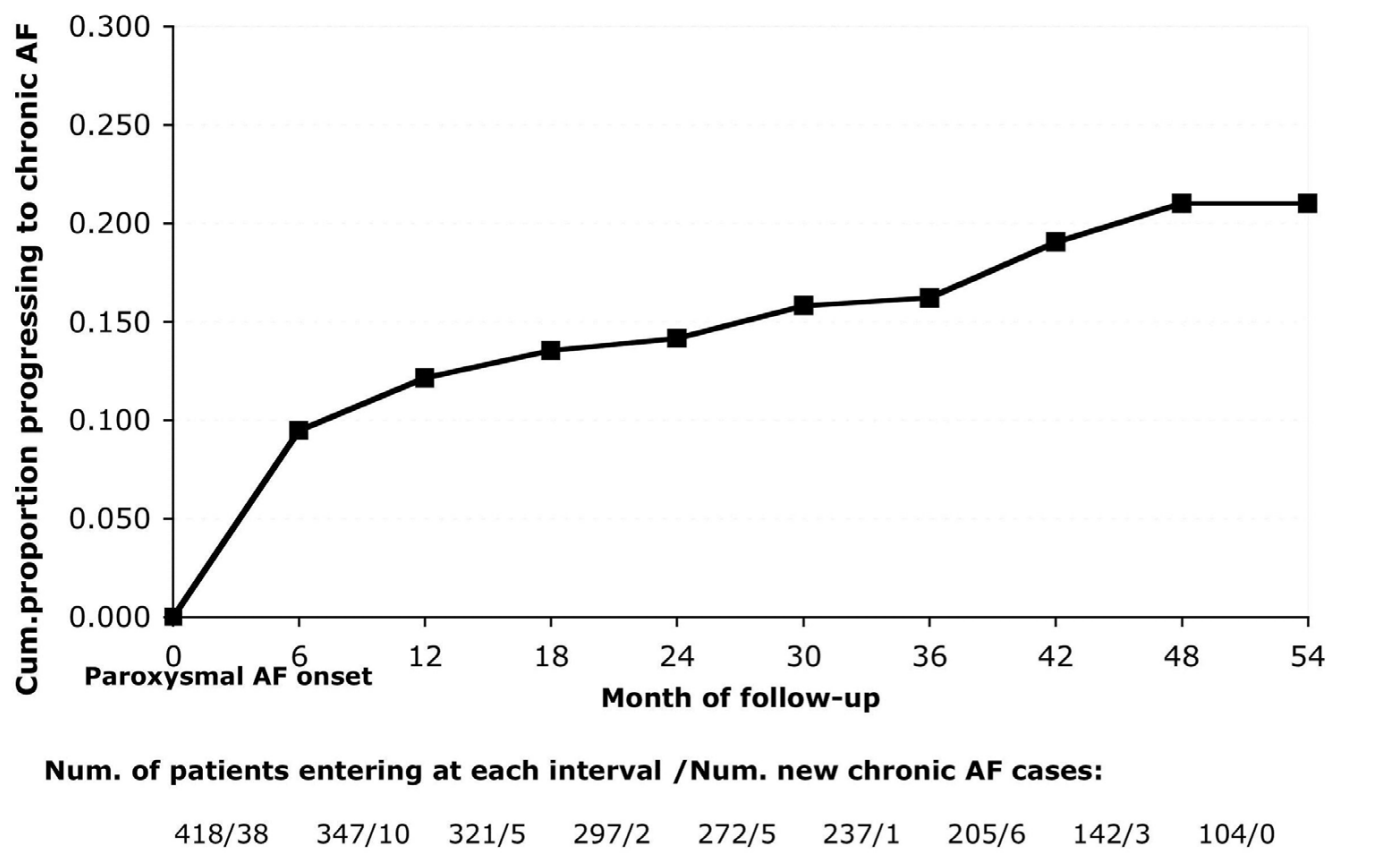
Proportion of patients with paroxysmal atrial fibrillation progressing to chronic AF. Number of patients at risk and number of new chronic AF cases in each time period, in UK General Practice.

Seventy patients with paroxysmal AF eventually progressed into chronic AF during the follow-up period. The progression rate was 6.2 per 100 person-years, with a slightly higher rate among men (6.8 per 100 person-years) than women (5.6 per 100 person-years). Fig. 2 shows the proportion of patients developing chronic AF over time, using life table analysis. It also present the number of patients with paroxysmal AF at risk and the number of patients progressing to chronic AF in each time interval. Patients dying or leaving the practice were censored from follow-up. More than half of the cases of progression (n = 48) occurred during the first year after the initially paroxysmal AF onset, resulting in a progression rate of 13.6 per 100 person-years during the first year of follow-up. Among those not progressing to chronic AF (n = 348), 156 had only the initial single episode recorded and 192 presented recurrent episodes of paroxysmal AF during follow-up.

**Figure 3:**
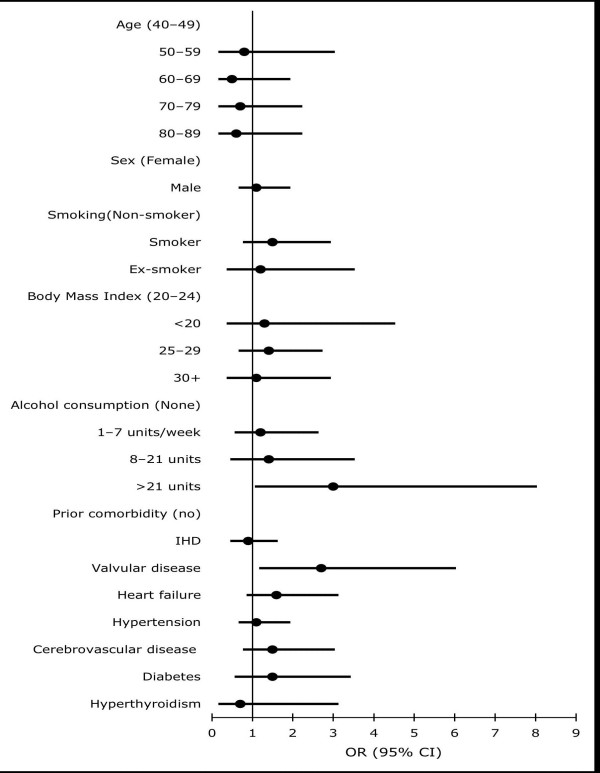
Risk of progression to chronic atrial fibrillation among paroxysmal atrial fibrillation patients (Odds ratio estimates adjusted by age and sex, using logistic regression).

Figure 3 presents the main characteristics studied as potential risk factors for the progression to chronic AF. Age and sex were not associated with progression to persistent AF. Only cardiac morbidity, mainly valvular disease and heart failure were associated with significantly greater risk of developing chronic AF. Moderate to high consumption of alcohol (more than 21 units per week) was associated with a three-fold increased risk of progression (fig. 3). We did not find any association between use of antiarrhytmic drugs after initial diagnosis of paroxysmal AF and progression or not to chronic forms of AF (data not shown), but we observed that patients progressing to persistent AF were more likely to have been treated with warfarin after their initial PAF diagnosis (OR: 2.9; 95%CI: 1.6–1.9) compared to those not progressing.

Eleven patients died among the patients progressing to chronic AF and 39 in the subgroup not progressing. The mortality rate was higher among patients progressing to chronic AF (5.2 per 100 person-years) than in the group who did not (3.6 per 100 person-years). The age- and sex-adjusted relative risk of mortality was 1.5 (95% CI 0.8–2.9) among patients who had progressed to sustained forms of AF, compared with those not progressing.

## Discussion

This is the first study, to our knowledge presenting follow-up data in patients initially diagnosed with paroxysmal atrial fibrillation from the perspective of a general practice setting, and we found the annual incidence of paroxysmal AF to be 1.0 per 1000 person-years. A slightly lower incidence rate (0.6 per 1000) has been reported in a study where paroxysmal AF patients attending hospital were identified [13]. The incidence of paroxysmal AF was almost half the one we reported for chronic AF in the same source population [10]. This is well in line with what has been reported previously – that paroxysmal episodes represent between 35% and 66% of all cases of AF, depending on the study population and the definitions used [1,3,14]. It has been reported that the load of patients with atrial fibrillation is likely to increase substantially in the next years, explained only in part by the aging of the population [15]. Our results show, as previously reported, that patients with paroxysmal AF are younger and have less comorbidity than patients with chronic AF [10,13,16]. This age difference could reflect the progressive nature of AF. The differences in morbidity and mortality among the two forms of AF suggest the possibility that paroxysmal and persistent AF may be different diseases with different risk factors and different pathogenic substrates, although clearly overlapping in part [17-19], paroxysmal being a more benign disease than chronic.

It is difficult to assess an underlying cause in all AF patients [3]. In our study, the specific cause was not reported in 32% of paroxysmal AF patients. We observed that the most frequent underlying associated conditions in paroxysmal AF were coronary heart disease, rheumatic heart disease and hypertension. These causes have also been found for chronic AF [12], although there appears to be a great variability between studies.

It has been recommended that pharmacological management of patients with newly discovered AF requires knowledge of its pattern of presentation (paroxysmal, persistent, or permanent) [4]. Anticoagulation with warfarin has been proposed for patients with paroxysmal forms when there is underlying heart disease [9,20-22], as no major differences in risk of stroke between chronic and paroxysmal AF have been reported. However it is not clear whether patients with limited episodes of paroxysmal AF require anticoagulation, and the decision must be individualized for each patient [4]. We found that close to half of the patients attending general practice with initial paroxysmal AF were not given warfarin or aspirin in the three months after the first-detected episode – a higher proportion than the one we observed among patients with chronic forms of AF [10]. This could be due in part to the fact that 37% of patients with paroxysmal AF presented a single episode of paroxysmal AF without any recurrence during the follow-up, and consequently anticoagulant or antiplatelet therapies may not be recommended in this subgroup of patients [19].

Clinical experience suggests that paroxysmal AF is often perpetuated and frequently it progresses to chronic AF, with estimates ranging between 20% and 30% within a period of 1–3 years [1,23,24]. A ten year follow-up study of paroxysmal patients in Trieste reported that 34% developed chronic AF [18]. The rate of progression varied according to aetiology, with patients with rheumatic valve disease carrying the highest rate of progression (66%) [4]. A recent 14 years follow up study on Japanese patients with initial paroxysmal AF, reported that 77% of them developed into its chronic form (5,5% of patients per year) [25]. In our study, we found that 17% of paroxysmal AF patients progressed to chronic AF during an average follow-up of close to 3 years, and history of valvular heart disease and moderate to high alcohol consumption were identified as the major independent predictors of progression to chronic AF.

Only a few small studies have examined factors predicting progression from paroxysmal to persistent AF [23,24]. The study by Abe et al. of 122 consecutive patients with paroxysmal AF reported a progression of 11% to chronic AF during a period of 2 years. They did not find any significant differences in age, sex or presence of organic heart diseases in the patients who developed chronic AF, compared with paroxysmal AF patients who did not progress[23]. Another study reported an annual progression rate of 22% [24]. Chronic and persistent forms of AF have been reported to carry a greater mortality than paroxysmal AF [17,23,24]. Our study suggests that patients progressing to chronic AF had a slightly higher increased risk of mortality than those not progressing.

Some limitations need to be taken into account when interpreting our results. Our findings are the reflection of detection and management of patients with newly diagnosed AF in general practice. Furthermore, our data are based on physicians assessments of patients symptoms and when available results from diagnostic tests to determine if the atrial fibrillation was paroxysmal or chronic (we could not specifically distinguish between permanent or persistent), and consequently will not be as accurate as prospective studies based on events detected after Holter monitoring, pacemaker insertion or ablation surgery. Our study was observational based on the information recorded by general practitioners during their daily practice. This shares the limitations of insufficient information at times but has the advantage to study the occurrence and determinants of AF patients in the real world of general practice. Therefore, it is likely that we may not have ascertained all cases of paroxysmal atrial fibrillation occurring in the study population, resulting in some underestimation of paroxymal AF. We found that a large proportion of patients had a unique episode of PAF, but we could not verify whether this was due to effective treatment with antiarrhythmic drugs or was incomplete reporting of subsequent paroxysmal episodes.

As we rely on the judgment of the GPs as well as their records and tests, some information was missing, for example GP could not assign a specific aetiology in 32% of paroxysmal AF patients, and we could not distinguish between those who would truly be "lone AF" and those with an underlying etiology not recorded by the GP. Also the rate of progression from paroxysmal to chronic AF could have been underestimated as we did not obtain complete response for all followed-up patients.

## Conclusion

In summary, we observed that paroxysmal AF is a common arrhythmia in a general practice setting, increases with age, and is commonly associated with other cardiac morbidity. Paroxysmal AF is sometimes the initial presentation of an arrhythmia progressing to chronic AF. Valvular heart disease and alcohol consumption are associated with this progression. Patients with paroxysmal AF do not present an increased risk of mortality compared to the general population after adjustment for other comorbidity, except among the subgroup progressing to chronic AF where a small mortality excess risk was observed.

## Competing interests

AR and LAGR work at CEIFE, which received a research grant from AstraZeneca for this study. MAW and SJ are employees and shareholders of AstraZeneca. The authors do not expect any companies to which they have provided services to gain or lose financially from the materials in this study.

## Authors' contributions

AR and LAGR conceived the study, participated in its design, performed the analysis and wrote the manuscript. MAW and SJ contributed to the review of patients profiles, data analysis and participated in the writing of the mansucript. All authors read and approved the final version of the manuscript.

## Pre-publication history

The pre-publication history for this paper can be accessed here:


